# An Update on Clinicopathological and Molecular Features of Plexiform Fibromyxoma

**DOI:** 10.1155/2019/3960920

**Published:** 2019-07-07

**Authors:** Hsuan-An Su, Hsu-Heng Yen, Chih-Jung Chen

**Affiliations:** ^1^Department of Medical Education, Kaohsiung Chang Gung Memorial Hospital, Kaohsiung, Taiwan; ^2^Endoscopy Center, Changhua Christian Hospital, Changhua, Taiwan; ^3^School of Medicine, Chung Shan Medical University, Taichung, Taiwan; ^4^Department of Surgical Pathology, Changhua Christian Hospital, Changhua, Taiwan; ^5^Department of Pathology and Laboratory Medicine, Taichung Veterans General Hospital, Taiwan

## Abstract

Plexiform fibromyxoma is a rare and newly described gastric mesenchymal tumor with only 121 reported cases in the literature. Our understanding of plexiform fibromyxoma requires updating since the first case has been reported by Takahashi et al. 12 years ago. The present review summarized reported cases in the literature, and both clinical and pathological aspects of plexiform fibromyxoma were comprehensively discussed. Plexiform fibromyxoma usually causes nonspecific or bleeding signs or symptoms, and therefore clinical recognition of the disease is challenging. Plexiform fibromyxoma is of benign nature without any metastasis or recurrence reported, and more conservative surgical treatment should be considered.

## 1. Introduction

Plexiform fibromyxoma (PF), also known as plexiform angiomyxoid myofibroblastic tumor (PAMT), was first described in 2007 by Takahashi et al. This rare tumor of mesenchymal origin is typically seen in the stomach and shows clinically benign behavior. As implied by the name, it features a plexiform cellular architecture of a myofibroblastic nature, encircled by a myxoid intercellular matrix with rich vasculature [1].

To date, only 121 cases have been reported in the literature with a designation of PF or PAMT; the clinical features of reported cases are summarized in Table 1. Specious case studies were published before the Takahashi's first report in 2007, terming the condition “gastric fibromyxoma,” “gastric myxoma,” or “fibromyxoangioma” [2–8], but these are not included in the present review because the diagnoses could not be ascertained by immunohistochemical staining despite the similarity of the clinical features. Many cases have been reported since Takahashi first defined the entity 12 years ago; however, as new cases emerge, many facts about PF require updating, including the terminology, epidemiological data, various clinical presentations, diagnosis, diverse strategy on treatment, and prognosis. Correct updated information will help in the clinical recognition of the disease and improve the outcomes of treatments. The aim of this review is to provide a comprehensive updating of PF including the published cases to summarize what we know about PF, to identify what we still need to investigate, and to achieve consensus on all aspects of this disease. Hence, we present a review of the published cases in the literature and discuss clinically significant issues about PF.

## 2. History and Nomenclature

Takahashi et al., in 2007, reported 2 cases of gastric plexiform fibromyxoma using the term “plexiform angiomyxoid myofibroblastic tumor (PAMT)” because the condition was morphologically distinct from other gastrointestinal mesenchymal tumors due to its bland spindle cells in a plexiform pattern, myxoid intercellular matrix with hypervascularity, and myofibroblastic nature [1]. In 2008, Yoshida et al. reported 2 additional cases and modified the designation to “plexiform angiomyxoid tumor,” deleting the term “myofibroblastic” due to the evident differentiation into smooth muscle cells that had been absent from the previous report and emphasizing the differentiating potential of myofibroblasts into smooth muscle cells. Yoshida et al. also further characterized the spectrum of fibrous, fibromyxoid, and myxoid stromal patterns of the disease [9]. Takahashi et al. continued to use the designation “myofibroblastic,” because this presentation was seen in the majority of cases [10].

In 2009, Miettinen et al. described 12 cases of the disease and designated the tumors as “plexiform fibromyxoma” simply due to their cellular architecture and fibromyxoid nature [11]. They also identified previously reported diseases from 1959 to 1986 that shared similar characteristics with PF by the names “gastric fibromyxoma” or “gastric myxoma” [2, 5, 6, 8, 12]. In 2010, Takahashi et al. argued that the designation “plexiform fibromyxoma” could lead to confusion, since “gastric fibromyxoma” or “gastric myxoma” describes a relatively narrow entity of purely fibroblastic tumors that present a different immunoprofile from that of PAMT; consequently, those cases reported as “gastric fibromyxoma” or “gastric myxoma” might actually differ from PAMT and would require further pathological or immunological evidence [10]. However, the WHO classification of tumors of the digestive system later adopted the term “plexiform fibromyxoma” in 2010 to designate the entity [13]. Since then, both “plexiform fibromyxoma” and “plexiform angiomyxoid myofibroblastic tumor” have both been used as the nomenclature of the disease, with controversy in the literature. Despite the nomenclature set by the WHO classification, many authors still preferred the term PAMT as a better description of both the histogenesis and histology of the tumor [14–16]. In the present review, 85 cases were designated as PF, while 36 were designated as PAMT.

Sing et al. proposed that PF and PAMT are two related, but different, entities at two respective fibroblastic and myofibroblastic ends of a spectrum [20]. Yet, due to the generally close similarity of the two, Duckworth et al. considered the two to be a single entity with an acceptable range of histologic, immunohistochemical, and ultrastructural presentations [34]. By contrast, Sing et al. argued that PF occupies the “fibroblastic” end while PAMT occupies the fully differentiated “myofibroblastic” end of the spectrum, and that the size, female exclusiveness of PF, vascular invasion, and extragastric extension are distinguishable between the two, despite the similar location of occurrence and age of onset [20]. Immunohistochemically, Sing et al. suggested that desmin and caldesmon could be used to distinguish PAMT as it shows focally positive results, whereas PF shows negative results. However, on the grounds of the limited case numbers for PF and PAMT and that desmin and caldesmon were not specific for the myofibroblastic form only, we suggest that discrimination of the two designations is unnecessary. In the literature, the cases designated as PF and those as PAMT showed no significant difference in age of onset or mean tumor size and both had female predominance and extragastric involvement. Although vascular invasion had only been reported in PF cases, the number of cases with vascular invasion is very small.

Since the WHO classification had designated the nomenclature PF for this entity, to avoid confusion in the literature, we suggest the use of PF in consideration of epidemiological surveillance and scientific communication purposes. The term PAMT literally describes the features of the disease in more detail, but PF could be viewed sensu lato as a broader nomenclature that covers the disease as well as other variants. We consider this to be acceptable, given that the disease is a spectrum with variations. The final designation or subsets under PF could be further categorized in the future after consideration of sufficient case numbers and advanced investigations.

## 3. Epidemiology

According to the 110 cases reported in the literature from 2007 to 2018, the frequency of PF is more than 9.17 cases per year worldwide. However, we feel this is an underestimate, because clinical recognition of the disease has only been increasing since 2007, and some cases are assumed to have been misidentified as gastrointestinal stromal tumors (GISTs) or other entities. Miettinen et al. estimated that the frequency of PF is 150 times lower than gastric GIST [11]; however, about 3,000 cases of gastric GIST are diagnosed yearly in the United States [79]. Therefore, we consider a proportion of 1:150 to be a rather conservative estimation and should be far smaller.

The patient ages showed a broad range, from 5 to 81 years (mean age 43.17±18.00 years; median age 46 years). Most patients were middle-aged, with a peak around 30–60 years old. This age distribution of PF was in approximate accordance with previous reviews [10, 51]; however, the adult-to-child ratio was 8:1 by our estimation, unlike the 5:1 ratio stated by Morris's [51] but more similar to the 7:1 ratio proposed by Fukuzawa's in a recent systematic review [75]. The previous studies all reported a 1:1 male-female ratio for PF [10, 51, 59], although we found a slight female predominance, with male patients accounting for 43% and female patients for 57%.

The races of patients were not provided in most of the studies; consequently, the countries of the studies were substituted for the ethnicity of the patients unless the ethnic information of the patients was given. In terms of regions, most cases were reported from East Asia (N=58; 47.9%), followed by North America (N=29; 24.0%), Europe (N=26; 21.5%), South-East Asia (N=4; 3.3%), South Asia (N=3; 2.5%), and Africa (N=1, 0.8%). In terms of countries, most cases were reported from China (N=41; 33.9%), followed by the United States (N=29; 24.0%), Belgium (N=17; 14.0%), Japan (N=10; 8.3%), and Korea (N=6; 5.0%). This distribution may not reflect the genuine epidemiological status of PF, since a higher frequency may result from a higher quality of healthcare and from a larger population. Nevertheless, it still suggests that PF is a worldwide disease with a predominance in East Asia.

## 4. Clinical Presentation

PF has a benign nature but is associated with hypervascularity; therefore, the clinical presentation can range from incidental findings to nonspecific gastrointestinal (GI) symptoms and further to severe GI hemorrhage. The typical presentation of PF is a nonspecific gastrointestinal complaint, such as abdominal pain, abdominal distension, and abdominal discomfort. Hemorrhagic gastrointestinal presentations are also commonly seen, with consequent GI bleeding-associated presentations like anemia, melena, and hematemesis [10, 36, 68, 74]. The available literature includes 121 cases, with clinical signs and symptoms available for 95 cases. The clinical presentations listed in Table 2 could be sorted into 3 categories, including abdominal signs or symptoms, bleeding signs or symptoms, and others. Most clinical presentations were abdominal signs or symptoms, mostly nonspecific, such as abdominal pain, abdominal distension, abdominal discomfort, abdominal mass, nausea, and heartburn. Many patients also presented with bleeding signs or symptoms, including anemia, melena, GI bleeding, and hematemesis. Severe hemorrhage leading to syncope [11, 53, 74] or hemodynamic instability [58] was also reported. Reflux symptoms might be present, with or without other complaints, and are likely to mask PF if presenting alone or with nonspecific complaints [22, 26, 68, 71, 80]. Among the cases that presented with reflux symptoms, only one tumor was located at the gastric fundus and might have been the cause of the symptoms [22]; other tumors were located at the gastric pylorus or antrum [26, 53, 68, 71, 80]. The likelihood that PF could directly or indirectly provoke reflux symptoms is doubtful, and the reflux symptoms were more likely to be concurrent with PF because of the high GERD prevalence worldwide. Ten cases were diagnosed incidentally, even with ulcerative PF lesions [25, 33, 59]; the size of incidentally diagnosed tumor ranged from 0.8 to 4.5 cm, and 4 tumors were located at gastric body, 1 was located at gastric fundus, and 5 were located at gastric antrum or pylorus [1, 16, 25, 27, 33, 37, 56, 59, 61]. Two cases with hemorrhagic perforation were reported by Takahashi et al. and Tan et al., with maximal tumor diameters of 4 and 3.5 cm, respectively [1, 21]. Other signs or symptoms at presentation included amenorrhea with cushingoid appearance in a 35-year-old female, but her symptoms were actually caused by polycystic ovary syndrome [20]. Chest pain with shortness of breath and finger numbness in a 16-year-old female was probably a result of a mass effect of the 3.2 cm PF in the mediastinum [34], while cholelithiasis was reported in a 55-year-old female with a PF in the gallbladder [42]. Fever was reported in a 42-year-old female with a fistulating abscess formation connecting the tumor and gastric lumen, suggesting possible infection sequelae of PF if left untreated [36]. The pathogenic association between these signs or symptoms and PF could not be proven; however, physicians are reminded of the nonspecific presentation of the disease. If a gastric neoplasm is clinically suspected, further endoscopic diagnostic intervention is indicated.

## 5. Location

Although initially categorized as a gastrointestinal mesenchymal tumor [1], PF has been reported to occur at locations other than in the gastrointestinal tract. The locations of the tumors were reported in 120 cases. Most of the tumor locations were the gastric antrum (including pylorus and gastric angle, N=95; 79.2%), followed, in decreasing order, by gastric body (N=10; 8.3%), stomach (inside location unspecified, N=5; 4.2%), gastric fundus (N=4; 3.3%), duodenum (N=2; 1.7%), jejunum (N=2; 1.7%), gallbladder (N=1; 0.8%), and mediastinum (N=1; 0.8%). The tumors often involve the pylorus and extended into the duodenal bulbs, probably causing obstruction [10, 11, 71]. Therefore, despite its gastric predominance, PF does not exclusively occur in the stomach and is also not confined to the GI tract, as indicated by 114 gastric tumors and 6 extragastric tumors.

## 6. Endoscopic Findings/Macroscopic Pathological Findings

The size of the tumors, available in 98 cases, ranged from 0.8 to 17 cm in the maximal diameter, with an average size of 4.81±3.30 cm and a median size of 4.0 cm. Endoscopic visualization reveals that PFs are typically pink or reddish and glistening tumors, elastic in texture, and covered with ulcerative, erosive, or smooth mucosa. Endoscopic ultrasonography indicates that PFs are hypoechoic with mild heterogenicity. Macroscopic examination shows a classical PF appearance as a lobulated tan-white or grayish-whitish mass, gelatinous on the cut surface, cystic, with mucinous fluids, a multinodular or polypoidal growth pattern, unencapsulated, and with well-defined (but sometimes ill-defined) margins. Hemorrhage is commonly encountered. PFs mostly originate from the submucosa and muscularis propria, with extension ranging from the mucosa to the serosa, causing ulcer and/or perforation.

The condition of the tumor surface was reported in 76 cases: 50 (65.8%) were ulcerated, 22 (28.9%) were nonulcerated, and 4 (5.3%) were covered with eroded mucosa. Ulceration or erosion of the tumor was significantly associated with hemorrhage-related signs or symptoms, as determined by Pearson's chi-squared test (*p*<0.0001). The difference in tumor size between ulcerative or erosive lesions and nonulcerative lesions was not statistically significant, as calculated by an independent Student's t-test (*p*=0.184).

## 7. Microscopic Findings

The signature of PF, as disclosed in the name PAMT, is the presence of bland ovoid to spindle cells arranged in irregular plexiform or multinodular pattern and separated by abundant myxoid and a variably collagenized extracellular background, interwoven with rich, arborizing, capillary-sized vasculature. The myxoid matrix is consistently Alcian blue positive. The tumor cells demonstrate monomorphous oval nuclei containing indistinct nucleoli and fine chromatin, surrounded by mildly eosinophilic cytoplasm with indistinct borders. Delicate and indistinct nucleoli and fine chromatin may be present. Cellular atypia and mitosis are both rare and are not seen in the majority of the cases. Microscopically, the tumor margin is infiltrative and unencapsulated; in 33 cases reporting the condition tumor margin, 20 were ill-defined and the rest were well-circumscribed. Necrosis was reported in the literature in only 2 cases: a 42-year-old female with fistulating abscess showed central necrosis as well as gas-fluids level [36], and a 31-year-old female with an ulcerative lesion exhibited only surface necrotic tissue coverage without central necrosis [74]. Some lymph nodes display enlargement with reactive changes [19, 67]. Vascular or lymphatic involvement was observed in 5 cases [11, 60].

In all, 118 cases reported immunohistochemical profiles of the tumor with various markers (Table 3). Immunohistochemical staining in most cases showed positive results for vimentin, smooth muscle actin (SMA), and muscle specific actin (MSA), indicating the fibroblastic, myofibroblastic, and smooth muscle cell natures of PF. Negative results for DOG-1, CD117 (KIT), CD34, S-100 protein, neurofilament, cytokeratin, epithelial membrane antigen (EMA), and ALK suggest that PF is a distinct disease entity from GIST, angiomyxoma, neurogenic tumor, sarcomatoid carcinoma, and inflammatory myofibroblastic tumor. Partial immunoreactive or focally positive results with desmin, caldesmon, and CD10, consistent with a partial or incomplete muscle immunophenotype, suggest possible myofibroblastic differentiation. Ki-67 staining commonly illustrates very low proliferation rates, mostly <2%, indicating a very low grade/indolent mesenchymal tumor; nevertheless, 5% [44, 74], 6% [25], 30% [77], and a vascular endothelial Ki-67 index up to 40% [49] have also been reported.

Significantly or diffusely reactive immunostainings included vimentin (100%), SMA (89.1%), and MSA (90%); however, these are nonspecific markers for mesenchymal and myofibroblastic lineages and were therefore sensitive but specific for diagnosing PF. Equivocal staining results were demonstrated for desmin, caldesmon, calponin, CD10, estrogen receptor (ER), and progesterone receptor (PR). Desmin and caldesmon are more specific markers for muscular lineage toward terminal muscle cell differentiation and exhibited limited and focal reactive results in PF, consistent with the proposed myofibroblastic spectrum of PF cell development.

Calponin is a nonspecific muscular marker for differentiated smooth muscle cells, while CD10 indicates cells with fibroblastic traits. The calponin and CD10 results confirmed the variably myofibroblastic nature of PF in the muscular and fibroblastic axes, respectively. The ER showed all negative staining, but the PR were diffusely or focally reactive in 8 of 10 cases [20, 31, 59], one of which revealed a prominent PR positivity in 80% of the tumor cells [20]. The PR positivity implied that PF may be sensitive to hormonal therapy [20, 59], but this might not be clinically practical. PF is reminiscent of extra-uterine or metastatic endometrial stromal sarcoma, which is rare but most commonly occurs in the GI tract [20]. However, it typically presents ER positivity [81], while PF consistently shows negative ER immunostaining result.

The rest of the markers mainly had negative results in PF. DOG-1 and CD117 were always negative in the reported cases, which allowed PF to be well-distinguished from GIST [82]. CD34 was mostly negative (95.1%), but focally positive results have been shown in several cases. CD34 could be labeled in cells with fibroblastic nature [83], but CD34 shows strong positive staining in vascular endothelial tissues; therefore, we suggest the possibility that focally positive or equivocal results originate from the rich vascularity of PF or as a result of technical or interpretation errors. The PF cases showed almost entirely negative results according to S-100 protein staining, indicating that PF is not derived from the neural crest; the 2 cases reporting positive results we highly suspect to be biased by technical error. Except for 1 case with a positive EMA staining result and 1 case with focally positive cytokeratin AE1/AE3 staining result, negative outcomes were reported for EMA, neurofilament, cytokeratin, *β*-catenin, ALK, cytokeratin AE1/AE3, and synaptophysin, thereby excluding epithelial, perineural, neuronal, and neuroendocrine cell lineage and ruling out some of the important differential diagnoses, such as fibromatosis and inflammatory myofibroblastic tumor.

Genetic mutation has also been examined in some studies. The* C-KIT *and* PDGFRA* gene mutations are important and characteristic in GIST [82], but they were both negative in all PF cases reported, thereby further enhancing the differentiation between PF and GIST. Genetic mutations involving* glioma-associated oncogene homologue 1 *(*GLI1*) and* metastasis-associated lung adenocarcinoma transcript 1* (*MALAT1*) have been identified in a subset of PF cases [54, 59], with* GLI1* gene translocation reported in 6 cases (24%) and* GLI1* polysomy reported in 2 cases (8%), out of overall 25 cases with* GLI1* genetic analysis [54, 59, 78]. The gene translocation t(11;12)(q11;q13) producing functional MALAT1-GLI1 chimeric proteins and the polysomy of GLI1/12q13 both will lead to overexpression of GLI1 protein. The overproduction of GLI1 protein has been recognized in a wide range of neoplasms [84, 85] and occurs via activation of the Hedgehog signaling pathway [54], which plays important roles in gastrointestinal developments, diseases, and neoplasms [86]. Apart from the canonical pathway of Hedgehog signaling, a noncanonical, Patched-dependent, and Smoothened-independent pathway has been recently described, which may be vital for the maintenance of gastrointestinal neoplasms [87]. The molecular pathway of PF development implicates that such neoplasms with* GLI1* oncogenesis may be sensitive to Hedgehog pathway inhibitors, targeting either Patched, Smoothened, GLI1, or other Hedgehog pathway components [88]. PF remains an emerging disease entity, and we are far from understanding its genetic profile with such a limited case number. Nevertheless, the present results of genetic analysis that aimed at benefiting differential diagnosis confirm a different genetic profile from GIST, and further research on the* GLI1* mutation and Hedgehog signaling may bring about improved understanding of its pathogenesis and even novel therapeutic strategies.

## 8. Diagnosis and Differential Diagnoses

Clinical findings are indecisive for the diagnosis of PF, as a solid, elastic tumor with or without ulceration is most likely to be encountered first during endoscopy, with typically nonspecific or hemorrhagic gastrointestinal signs or symptoms. Diagnosis of PF depends on pathological and immunochemical examinations, which are not achieved by clinical examination alone [73], and a minimum of three microscopic signatures should be observed, including spindle or oval cells in a plexiform growth pattern, rich and small-sized vasculature, and abundant myxoid matrix. The benignity of PF could be speculated by its limited atypia and low mitotic rate [89]. The plexiform pattern is highly characteristic of PF, so the designation of a tumor without plexiform growth pattern as PF is highly unlikely. Immunohistochemically, PF is almost always positive for vimentin and SMA and variably positive for desmin, caldesmon, and CD10. Negativities for DOG-1, CD117, CD34, S-100 protein, EMA, and ALK should be examined to exclude similar mimicking conditions.

The most important differential diagnosis of PF is GIST, especially myxoid variants of GIST with spindle-cell histology [77, 89]. Most PF cases were initially treated based on a suspicion of GIST, which is the most common gastrointestinal mesenchymal tumor and potentially fatal [77, 82]. Microscopically, although not totally impossible [89], a plexiform pattern is highly unusual for GIST [9]. In addition, the characteristic immunoprofiles of CD117, CD34, and DOG-1 immunoreactivities and* C-KIT* or* PDGFRA* mutations of GIST are adopted widely in routine pathological practice [82].

Other pathological differential diagnoses, based on spindle cell morphology and myxoid stromal background, include sarcomatoid carcinoma, peripheral nerve sheath tumor, inflammatory fibroid polyp, and inflammatory myofibroblastic tumor. Primary or secondary sarcomatoid carcinoma with spindle tumor cells and myxoid stroma could be sometimes mistaken as gastrointestinal mesenchymal neoplasm. A clinical cancer history, a more malignant cytological feature, such as brisk mitotic or apoptotic activities, positive epithelial immunoreactivities, and markers for possible metastatic origins, such as TTF-1 for pulmonary origin, are significantly conducive to the correct diagnosis [90].

Peripheral nerve sheath tumor includes benign schwannoma, neurofibroma or ganglioneuroma, and malignant peripheral nerve sheath tumor (MPNST); some variants also demonstrate a plexiform growth pattern [89]. Neurogenic markers, such as S-100 protein, SOX10, and neurofilament, highlight nerve sheath differentiation that is absent in PF [91, 92].

Inflammatory fibroid polyps consist of bland-looking spindle cells arranged in perivascular whorled pattern in a myxoedematous inflammatory stroma with mostly eosinophils. Positive CD34 immunoreactivity is the key characteristic of this rare tumor, which should be negative in PF except for vascular endothelial cells [93].

Inflammatory myofibroblastic tumor is characterized by monotonous spindle cells arranged in fascicles or vague whorls in an inflammatory and edematous stroma full of neutrophils, eosinophils, or lymphoplasma cells. Although the myofibroblastic nature of an inflammatory myofibroblastic tumor is similar to that of PF by morphology and immunohistochemical studies, the lack of a plexiform growth pattern, a predominantly inflammatory cellular microenvironment, and positive ALK immunoreactivity help to distinguish inflammatory myofibroblastic tumor from PF [94].

## 9. Treatment

The mainstream treatment of PF is surgical removal, while medical treatment serves an assistant role for symptomatic management. Among the 121 reported cases, treatments were available in 99 cases and not provided in 22 cases. Partial gastrectomy (N=30) and distal gastrectomy (N=26) are the most frequently performed surgical treatments for PF, followed by local resection (N=12), subtotal gastrectomy (N=10), wedge resection (N=7), submucosal dissection (N=6), antrectomy (N=3), partial duodenectomy (N=2), nonsurgical follow-up with only endoscopic biopsy (N=2), total gastrectomy (N=1), and laparoscopic cholecystectomy (N=1). A laparoscopic operative technique was performed in 10 cases, endoscopic resection in 9 cases (including 1 thoracoscopic resection), and laparoscopic endoscopic cooperative surgery (LECS) in 4 cases. None of the surgically treated cases presented with severe postoperative complications, except for a 63-year-old male patient who had experienced recurrent GI bleeding for 17 years with a duodenal PF and had undergone pancreas-preserving duodenectomy of the first 2 portions, complicated by a low output pancreatic-skin fistula which had totally drained after 4 months. Secondary procedures included Billroth's operation I (N=3), Billroth's operation II (N=2), Roux-en-Y anastomosis (N=2), and omentectomy (N=1).

Conservative management included endoscopic resection and nonsurgical intervention. Since PF is currently considered a benign disease (though not yet verified by convincing evidence), conservative management may be suitable for PF patients, and particularly for the elderly or selected patients with surgically contraindicated comorbidities [72]; at least, more conservative surgical management than partial gastrectomy could be feasible [23]. However, vascular and lymphatic invasion were reported by Miettinen et al. and Kawara et al. [11, 60], and PF usually develops in a nodular and plexiform pattern with unclear tumor margins [1, 30, 64], suggesting that the possibility of malignancy or local recurrence cannot be fully excluded [9, 73]. Therefore, regardless of the operative technique, we suggest that complete resection still be the first consideration when treating PF rather than consideration of the conservativeness of the operative technique [30, 64, 71]. PF cases with severe clinical presentations, such as perforation [1, 21], infection [36], or considerable hemorrhage [53, 58, 63, 74], and with malignant suspicion, such as significant body weight loss [11, 72] or rapid tumor growth [16], should be addressed with aggressive radical surgical treatment rather than conservative management, despite the benign pathological results.

## 10. Prognosis

PF shows a benign biological behavior [18, 73, 74], with a low proliferation rate and low mitotic rate, and no local recurrence or distant metastasis has been reported so far [74]. However, no consensus has been reached whether PF is actually a benign tumor, and no cases have confirmed that malignant change does not occur, so confirmation of the benign nature of PF still requires longitudinal observational studies with sufficient case numbers. Nevertheless, for the time being, PF should be considered a benign tumor, in response to the strategy of conservative management.

Of all the 121 reported cases, follow-up was not reported in 37 cases, and the follow-up period was not reported in 4 cases. No malignant change, local recurrence, or PF-related mortality was reported. Most of the cases were uneventful after treatments, and among the cases that had follow-up periods, the uneventful or alive duration ranged from 0.75 to 396 months, with an average of 44.29±72.5 months and a median of 15 months. An 81-year-old female who underwent nonsurgical management also had her symptoms resolved 3 months after [72], and a 45-year-old male in Lai's study who underwent only endoscopic biopsy without further resection also led an uneventful life at 0.2-year follow-up [77]. Three cases were reported to have died for unknown causes at 2 months, 14.5 years, and 25.5 years after diagnosis [11]. Vascular or lymphatic invasion was noted in 5 cases, none of which had adverse significance on prognosis [11, 60].

## 11. Conclusion

PF is a rare mesenchymal tumor with increasing clinical attention and occurs mostly but not exclusively in the GI tract. Typical clinical presentations are nonspecific GI signs or symptoms, or upper GI bleeding. Endoscopic biopsy is recommended for visualizing the microscopic features of PF with benign cytological traits, and immunohistochemical staining is required for diagnosis as well as for exclusion of GIST. Surgical local excision is the main treatment; however, more conservative management is suggested within an individually reasonable range because of its very good prognosis.

## Figures and Tables

**Table 1:** Clinical features of cases reported as PF or PAMT in the literature.

No	Year	Author	Population	Age	Sex	Clinical Presentation	Ulcer	Location	Size (cm)	Diagnosis	Treatment	Prognosis
1[1]	2007	Takahashi	Japan	50	M	acute abdominal pain (perforation)	+	pyloric antrum	4 × 4 × 2.5	PAMT	distal gastrectomy	NA

2[1]	2007	Takahashi	Japan	68	M	incidental	-	pyloric antrum	4.5 × 3.5 × 3.0	PAMT	partial gastrectomy	uneventful for 12 months

3[17]	2008	Galant	Belgium	61	M	hematemesis	erosion	antrum	3.7	PAMT	partial gastrectomy	uneventful for 6 months

4[18]	2008	Rau	Germany	50	F	morning nausea	+	antrum	1.9 × 1.8 × 0.8	PAMT	Median laparotomy and local excision	No recurrence for 3 months

5[9]	2008	Yoshida	USA	19	F	mass in the stomach	-	antrum	4.5 × 3.5 × 3.0	PAMT	distal gastrectomy	uneventful for 9 months

6[9]	2008	Yoshida	USA	46	M	upper gastrointestinal bleeding	+	antrum	3.5	PAMT	distal gastrectomy	uneventful for 4 months

7[11]	2009	Miettinen	USA	38	F	upper gastrointestinal bleeding, ulcer	+	antrum, lesser curvature	3 × 2	PF	distal gastrectomy	lost f/u

8[11]	2009	Miettinen	USA	62	M	progressive weight loss for months	-	antrum, pyloric	4 × 4	PF	partial gastrectomy, omentectomy	lost f/u

9[11]	2009	Miettinen	USA	75	F	unknown	+	antrum, serosa	5	PF	subtotal gastrectomy	died of unknown cause, 2 months

10[11]	2009	Miettinen	USA	65	F	weight loss, gastric ulcer	+	antrum, lesser curvature, duodenal bulb	5 × 4.5 × 2.5	PF	partial gastrectomy	died of unknown cause, 14.5 years

11[11]	2009	Miettinen	USA	33	M	anemia, weakness	+	antrum, anterior wall	5.5 × 3.5 × 3.5	PF	50% distal gastrectomy	alive without disease for 19.7 years

12[11]	2009	Miettinen	USA	43	M	gastrointestinal bleeding	+	antrum, near pylorus	5.5 × 4.5 × 4.5	PF	partial gastrectomy	alive without disease for 18.4 years

13[11]	2009	Miettinen	USA	56	F	unknown	-	pylorus, duodenal bulb	5.5+3	PF	partial gastrectomy	alive without disease for 19.9 years

14[11]	2009	Miettinen	USA	50	M	gastric outlet obstruction	-	antrum, duodenal bulb	7 × 6 × 6	PF	distal gastrectomy	died of unknown cause, 25.5 years

15[11]	2009	Miettinen	USA	21	M	syncope, anemia	+	antrum, prepyloric, duodenal bulb	9 × 6 × 5	PF	antrectomy, B1-reconstruction	alive with unknown status for 22 years

16[11]	2009	Miettinen	USA	16	F	hematemesis	+	antrum, pylorus	10 × 9 × 6	PF	distal gastrectomy	alive with unknown status for 3 years

17[11]	2009	Miettinen	USA	30	F	nonhealing gastric ulcer	+	antrum	10 × 9 × 6	PF	distal gastrectomy	alive without disease for 9 years, alive with unknown status for 24 years

18[11]	2009	Miettinen	USA	7	F	emesis, diarrhea, protruding abdominal mass	-	antrum, pylorus, duodenal bulb	15 × 11 × 8	PF	excision of tumor, gastric wall resection at the tumor attachment	lost f/u

19[19]	2009	Pailoor	Malaysia	23	F	melena	+	antrum	8.0 × 4.0	PAMT	partial gastrectomy	uneventful for 2 months

20[20]	2010	Sing	South Africa (Indian)	35	F	cushingoid appearance, amenorrhea	-	pyloro-antral region, anterior wall of the greater curvature	4 × 3 × 2	PAMT	exploratory laparotomy and wide local excision	uneventful for 12 months

21[10]	2010	Takahashi	Japan	23	M	abdominal pain, abdominal discomfort, melena	+	antrum, duodenal bulb	14 × 14 × 7	PAMT	partial gastrectomy	uneventful for 12 months

22[21]	2010	Tan	Australia (Filipino)	34	M	abdominal mass, abdominal discomfort, decreased appetite (perforation)	+	antrum	3.5 × 3.4 × 2.5	PAMT	distal gastrectomy	uneventful for 2 months

23[22]	2010	Wang	China	54	F	abdominal distension, heartburn, hiccup, regurgitation, loss of appetite	erosion	fundus	1.5 × 1.2	PAMT	endoscopic resection	uneventful for 6 months

24[23]	2010	Cooper	USA	64	M	epigastric pain	NA	antrum	4.0 × 2.0	PAMT	laparoscopic excision	uneventful for 3 years

25[24]	2010	Cui	China	54	F	abdominal distension, decreased appetite	erosion	fundus	0.8	PAMT	NA	NA

26[14]	2011	Kim	Korea	52	M	dyspepsia	+	antrum	3.5 × 2.3	PAMT	laparoscopic wedge resection	uneventful for 5 months

27[25]	2012	Kang	Korea	47	M	incidental	+	mid body, posterior wall and greater curvature	3 × 2	PAMT	wedge resection	uneventful for 6 years

28[25]	2012	Kang	Korea	63	F	incidental	+	lower body, greater curvature	2.2 × 1.6	PAMT	endoscopic resection	uneventful for 1 month

29[26]	2012	Schulz	Germany	59	M	heartburn, upper abdominal pain	+	pylorus	1.5	PAMT	combined laparoscopic endoscopic local resection	NA

30[27]	2012	Cai	China	32	M	incidental	NA	antrum		PF	distal gastrectomy	NA

31[27]	2012	Cai	China	47	F	upper abdominal pain	NA	antrum		PF	radical distal gastrectomy	NA

32[28]	2012	Wang	China	12	M	gastrointestinal bleeding	NA	antrum		PF	partial gastrectomy	uneventful for 84 months

33[29]	2012	Miettinen	USA	49	F	NA	NA	antrum	3.5 × 2.5 × 2.5	PF	wedge resection	NA

34[30]	2012	Li	China	47	F	epigastric discomfort, abdominal pain	-	antrum	5 × 3 × 2	PAMT	laparoscopic distal gastrectomy	uneventful for 1.5 years

35[31]	2012	Bi	China	31	M	Abdominal pain	-	Antrum	8.0	PAMT	NA	NA

36[31]	2012	Bi	China	47	F	Abdominal pain	+	Antrum	4.5	PAMT	NA	NA

37[31]	2012	Bi	China	42	F	Abdominal distension	-	Antrum	4.6	PAMT	NA	NA

38[32]	2013	Kao	China	53	F	Abdominal distension, abdominal pain, nausea, acid regurgitation	NA	NA	NA	PAMT	NA	NA

39[33]	2014	Baek	Korea	38	F	incidental	+	upper body, posterior wall and greater curvature	3.5 × 2.3	PAMT	wedge resection	uneventful for 6 months

40[34]	2014	Duckworth	USA	16	F	chest pain, shortness of breath, finger numbness	NA	posterior mediastinum near the esophagus at the level of the carina	3.2	PF	thoracoscopic resection	uneventful for 14 months

41[34]	2014	Duckworth	USA	11	F	severe iron-deficiency anemia	+	pylorus	3.5	PF	laparoscopic distal gastrectomy	uneventful for 15 months

42[35]	2014	Ikemura	Japan	27	F	epigastric pain, melena, anemia	+	antrum of the lesser curvature side	4.6 × 3.0 × 2.8	PAMT	partial gastrectomy	uneventful for 40 months

43[36]	2014	Lee	Hong Kong (Filipino)	42	F	abdominal pain, fever, anemia (fistulating abscess formation)	+	antrum	12.9 × 11.9 × 10.6	PF	distal gastrectomy	uneventful for 3 weeks

44[37]	2014	Li	Chinese	32	M	incidental	-	antrum, anterior wall	3.4 × 3.0	PAMT	partial gastrectomy	uneventful for 3 years

45[38]	2014	Sakamoto	Japan	60	M	epigastralgia	+	antrum	2	PF	laparoscopic partial gastrectomy	uneventful for 12 years

46[39]	2014	Li	China	73	F	upper abdominal pain	NA	antrum		PF	partial gastrectomy	NA

47[40]	2014	Tian	China	64	M	upper abdominal discomfort	-	antrum	3.3 × 2.5	PF	distal gastrectomy	uneventful for 6 months

48[41]	2015	Banerjee	India	19	F	dull upper abdominal pain, lump in the right hypochondrium	+	duodenum (D1), posterior	13.8 × 8.6	PAMT	distal gastrectomy, proximal duodenectomy, Billroth II gastrojejunostomy	uneventful for 6 months

49[42]	2015	Fassan	Italy (Caucasian)	55	F	cholelithiasis	NA	gallbladder	1	PF	laparoscopic cholecystectomy	no postsurgical complication

50[43]	2015	Lu	China	26	F	progressive abdominal distension, nausea, vomiting, melena	NA	antrum	NA	PAMT	distal gastrectomy	NA

51[44]	2015	Ni	China	21	F	melena, dizziness, anemia	+	antrum, greater curvature	4 × 3 × 3	PF	distal gastrectomy	uneventful for 3 years

52[45]	2015	Wei	China	50	F	upper abdominal pain	NA	antrum		PF	subtotal gastrectomy	uneventful for 3 months

53[46]	2015	Yue	China	34	M	upper abdominal pain	NA	antrum		PF	distal gastrectomy	uneventful for 24 months

54[46]	2015	Yue	China	50	F	upper abdominal pain	NA	antrum		PF	subtotal gastrectomy	uneventful for 3 months

55[47]	2015	Xu	China	50	F	abdominal pain	NA	gastric body	3 × 2	PAMT	Total gastrectomy	uneventful for 6 months

56[15]	2016	Dixit	India	51	F	abdominal pain, vomiting, weight loss (with synchronous ovarian neoplasms)	+	pylorus	8.4 × 6.1 × 5	PAMT	distal gastrectomy	no postsurgical complication

57[48]	2016	Inoue	Japan	36	F	epigastric pain, anemia	+	antrum	2.5 × 2.2 × 2.0	PF	radical resection with laparoscopic endoscopic cooperative surgery	uneventful for unknown period

58[49]	2016	Jonaitis	Lithuania (Caucasian)	28	F	epigastric pain associated with meals, iron-deficiency anemia, weight loss	+	antrum, anterior wall	3	PAMT	partial gastrectomy of the Billroth I type	follow-up not reported; gastroduodenal anastomositis, resolved with conservative treatment

59[50]	2016	Kane	USA (Vietnamese)	28	F	acute, severe abdominal pain and worsening anemia	+	antrum	5.5 × 3.5	PF	distal gastrectomy with a Roux-en-Y gastrojejunostomy	dyspepsia 23 months thereafter, otherwise uneventful for 23 months (only moderate stenosis of anastomosis)

60[51]	2016	Morris	USA	9	F	intermittent abdominal pain, nausea, vomiting, weight loss	+	antrum, posterior	5	PF	laparotomic anterior gastrotomy with tumor resection	uneventful for 6 months

61[52]	2016	Nagahisa	Japan	39	M	epigastric pain	+	antrum, anterior wall	3.5 × 3	PAMT	partial gastrectomy with laparoscopic endoscopic cooperative surgery	uneventful for 9 months

62[53]	2016	Quero	Italy	47	M	syncope, regurgitation, epigastric discomfort	+	antrum	6.5	PAMT	distal gastrectomy	uneventful for 10 months

63[54]	2016	Spans	Belgium	19	M	NA	NA	antrum	5.5	PF	NA	NA

64[54]	2016	Spans	Belgium	65	F	NA	NA	antrum	4.3 × 3.0 × 1.7	PF	NA	NA

65[54]	2016	Spans	Belgium	58	F	NA	NA	antrum	NA	PF	NA	NA

66[54]	2016	Spans	Belgium	51	F	NA	NA	antrum	9.0 × 8.5 × 5.5	PF	NA	NA

67[54]	2016	Spans	Belgium	63	F	NA	NA	jejunum, proximal	3.5 × 3.0 × 2.0	PF	NA	NA

68[54]	2016	Spans	Belgium	76	F	NA	NA	stomach	4	PF	NA	NA

69[54]	2016	Spans	Belgium	62	F	NA	NA	stomach	NA	PF	NA	NA

70[54]	2016	Spans	Belgium	30	F	NA	NA	stomach	2	PF	NA	NA

71[54]	2016	Spans	Belgium	28	M	NA	NA	gastric body	10 × 6 × 3	PF	NA	NA

72[54]	2016	Spans	Belgium	44	F	NA	NA	stomach	NA	PF	NA	NA

73[54]	2016	Spans	Belgium	18	F	NA	NA	antrum	4.5 × 3.5 × 2.7	PF	NA	NA

74[54]	2016	Spans	Belgium	19	F	NA	NA	stomach	4.5	PF	NA	NA

75[54]	2016	Spans	Belgium	46	M	NA	NA	antrum	3.5	PF	NA	NA

76[54]	2016	Spans	Belgium	29	M	NA	NA	antrum	6.5 × 4.5 × 4.0	PF	NA	NA

77[54]	2016	Spans	Belgium	36	F	NA	NA	antrum	8	PF	NA	NA

78[54]	2016	Spans	Belgium	47	F	NA	NA	antrum	4.5 × 3.7	PF	NA	NA

79[55]	2016	Li	China	11	M	upper abdominal discomfort	NA	antrum		PF	partial gastrectomy	uneventful for 12 months

80[56]	2016	Li	Chinese	44	F	incidental	-	antrum	0.8 × 0.8	PAMT	endoscopic submucosal dissection	uneventful for 6 months

81[57]	2016	Zhang	China	48	M	upper abdominal pain	NA	antrum		PF	partial gastrectomy	uneventful for 12 months

82[16]	2017	Akai	Japan	55	M	incidental	NA	gastric angle	1.7	PAMT	laparoscopic partial gastrectomy	NA

83[58]	2017	Gonzalez-Cordero	Spain	37	M	upper gastrointestinal bleeding with hemodynamic instability	NA	antrum	5.8 × 5 × 4	PF	antrectomy	uneventful postoperatively

84[59]	2017	Hu	China	26	M	abdominal distension	+	fundus	1.8	PF	endoscopic submucosal dissection	uneventful for 32 months

85[59]	2017	Hu	China	31	F	abdominal distension	-	antrum	2.5	PF	endoscopic submucosal dissection	NA

86[59]	2017	Hu	China	72	F	abdominal distension	+	antrum	7	PF	distal subtotal gastrectomy	uneventful for 56 months

87[59]	2017	Hu	China	59	F	abdominal distension	+	antrum	2.5	PF	distal subtotal gastrectomy	uneventful for 68 months

88[59]	2017	Hu	China	52	M	incidental	+	fundus	1.2	PF	endoscopic submucosal dissection	uneventful for 24 months

89[59]	2017	Hu	China	59	F	melena, anemia	+	antrum	3	PF	distal subtotal gastrectomy	uneventful for 40 months

90[59]	2017	Hu	China	48	M	abdominal distension	+	antrum	3	PF	distal subtotal gastrectomy	uneventful for 95 months

91[59]	2017	Hu	China	58	M	abdominal pain	+	antrum	3.5	PF	distal subtotal gastrectomy	uneventful for 34 months

92[59]	2017	Hu	China	46	M	abdominal distension	-	antrum	4	PF	distal subtotal gastrectomy	uneventful for 65 months

93[59]	2017	Hu	China	40	F	melena, anemia	+	antrum	3.8	PF	distal subtotal gastrectomy	uneventful for 70 months

94[60]	2017	Kawara	Japan	66	M	gastric tumor	-	antrum	4 × 3	PF	endoscopic submucosal dissection	uneventful for 12 months

95[61]	2017	Kim	Korea	51	M	incidental	-	antrum, lesser curvature	2.0 × 1.5	PAMT	laparoscopic gastric wedge resection	uneventful for 3 years

96[62]	2017	Liang	China	11	M	right epigastric discomfort with episodic pain	-	pylorus, anterior	17 × 10.5 × 5	PAMT	partial gastrectomy	uneventful for 12 months

97[63]	2017	Moris	USA	63	M	upper gastrointestinal bleeding, anemia, melena	NA	duodenum	3 × 3	PF	pancreas-preserving duodenectomy of the first 2 portions	uneventful for 20 months, complicated only by a low output pancreatic skin fistula totally drained after 4 months

98[64]	2017	Qi	China	42	M	melena	+	antrum, greater curvature	3 × 2 × 1.8	PF	laparoscopic partial gastrectomy	uneventful for 12 months

99[64]	2017	Qi	China	66	M	epigastric pain	-	antrum, greater curvature	2 × 1 × 0.7	PF	endoscopic submucosal dissection	uneventful for 12 months

100[65]	2017	Szurian	Austria (Caucasian)	16	F	anemia, nausea	+	antrum, anterior wall	6.5	PF	distal gastrectomy and retrocolic gastrojejunostomy (Billroth II)	pulmonary embolism postoperatively and discharged on day 9, otherwise uneventful for 6 months

101[65]	2017	Szurian	Austria	34	M	epigastric discomfort, flatulence	+	pylorus	1.6	PF	distal gastrectomy and retrocolic gastrojejunostomy (Billroth II)	uneventful for 16 months

102[66]	2017	Wambura	Tanzania (Tanzanian)	41	F	anemia, epigastric discomfort, melena	+	antrum	5.5 × 5	PF	distal gastrectomy with Roux-en-Y gastrojejunostomy	uneventful for 12 months

103[67]	2017	Yang	China	50	F	abdominal pain	-	gastric upper body	15 × 10	PF	laparoscopic partial gastrectomy	uneventful for 2 years

104[68]	2017	Zhou	China	51	M	epigastric discomfort, heartburn	NA	antrum, greater curvature	4 × 3 × 1.8	PF	total resection	uneventful for 15 months

105[69]	2017	Wang	China	66	F	Abdominal pain, melena, dizziness, abdominal distension	erosion	Antrum	3	PF	NA	NA

106[70]	2018	Djurić	Serbia	14	M	iron-deficiency anemia, fatigue, epigastric pain	NA	Antrum	NA	PF	partial gastrectomy	NA

107[71]	2018	Jang	Korea	47	F	heartburn	-	pylorus	2.5 × 2.0	PF	antrectomy with Billroth I anastomosis	uneventful for 7 months

108[72]	2018	Rohit	USA	81	F	weight loss of 3.6 kg in 3 months	NA	gastric body, lesser curvature	2.7 × 2.4	PF	non-surgical follow-up	symptom resolved 3 months after

109[73]	2018	Wang	Chinese	52	F	upper abdominal pain	+	mid gastric body, greater curvature	1.5 × 1.0 × 1.0	PF	gastroscope-assisted laparoscopic wedge resection	uneventful for 10 months

110[74]	2018	Zhang	China	31	F	hematochezia, syncope, upper gastrointestinal bleeding, anemia	+	upper segment of jejunum	1.2 × 1.0	PF	exploratory laparotomy and resection of the upper jejunal tumor and local intestine	uneventful for 3 years

111	2019	Wu	Taiwan (Taiwanese)	59	F	acid regurgitation	-	antrum	2 × 1.2 × 1.2	PF	partial gastrectomy	uneventful for 20 months

112[75]	2019	Fukazawa	Japan	14	F	abdominal pain, hematemesis	+	antrum		PF	partial gastrectomy	uneventful for 16 months

113[76]	2019	Banerjee	USA	65	M	anemia, early satiety, dyspepsia, hematemesis	+	antrum	5 × 2.3	PF	wedge resection	NA

114[77]	2019	Lai	USA	21	F	NA	NA	gastric body		PF	partial gastrectomy	4 years

115[77]	2019	Lai	USA	42	F	NA	+	antrum		PF	partial gastrectomy	4 years

116[77]	2019	Lai	USA	79	F	NA	NA	antrum		PF	partial gastrectomy	1 year

117[77]	2019	Lai	USA	33	M	NA	NA	antrum		PF	partial gastrectomy	11 years

118[77]	2019	Lai	USA	60	M	NA	NA	antrum		PF	partial gastrectomy	1 year

119[77]	2019	Lai	USA	77	M	NA	NA	gastric body		PF	partial gastrectomy	2 years

120[77]	2019	Lai	USA	45	M	NA	NA	antrum		PF	only endoscopic biopsy	0.2 years

121[78]	2019	Li	China	5	M	pale complexion	NA	antrum	8.2 × 7.5 × 5.5	PF	distal gastrectomy	36 months

**Table 2:** Reported clinical presentations of PF in the literature.

Clinical Presentation	Count	Percentage (%)
*Abdominal Signs or Symptoms*		

Abdominal pain	33	20.6
Abdominal distension	13	8.1
Abdominal discomfort	10	6.3
Abdominal mass	5	3.1
Nausea	5	3.1
Heartburning sensation	4	2.5
Acid regurgitation	4	2.5
Vomiting	4	2.5
Decreased appetite	3	1.9
Dyspepsia	2	1.3
Diarrhea	1	0.6
Early satiety	1	0.6
Gastric outlet obstruction	1	0.6
Hiccup	1	0.6

*Bleeding Signs or Symptoms*		

Anemia	18	11.3
Melena	11	6.9
Gastrointestinal bleeding	7	4.4
Hematemesis	4	2.5
Syncope	3	1.9
Dizziness	2	1.3
Gastric ulcer	2	1.3
Fatigue	1	0.6
Hematochezia	1	0.6

*Others*		

Incidental	10	6.3
Weight loss	7	4.4
Amenorrhea	1	0.6
Chest pain	1	0.6
Cholelithiasis	1	0.6
Cushingoid appearance	1	0.6
Fever	1	0.6
Finger numbness	1	0.6
Shortness of breath	1	0.6

**Table 3:** Pathological and immunohistochemical profiles of PF cases reported in the literature.

No	Year	Author	Age	Sex	Ki-67	Vim	SMA	MSA	DOG-1	CD117	CD34	Desmin	Cald	S-100 protein	Calp	EMA	NF	CK	ER	PR	CD10	*β*-C	ALK	AE1/AE3	SNP	C-KIT	PDFGRA
1	2007	Takahashi	50	M	<1%	+	+	+		-	-	-	+/-	-			-	-				-				-	-

2	2007	Takahashi	68	M	<1%	+	+	+		-	-	-	+/-	-			-	-				-				-	-

3	2008	Galant	61	M		+	+			-	-	-		-		-											

4	2008	Rau	50	F	2%		+	+		-	+/- (focal)	+/- (sporadic)		-								-	-			-	-

5	2008	Yoshida	19	F						-	-			-		-			-	-	-					-	-

6	2008	Yoshida	46	M	<1%		+	+		-	-	+/- (focal)	+ (partial)	-	+ (partial)									-		-	-

7	2009	Miettinen	38	F			+ (8/10)		-	-	-	-	- (3/3)	-							+ (1/3), -(2/3)			- (3/3)		wt (3/3)	wt (3/3)
8	2009	Miettinen	62	M					-	-	-	-		-													
9	2009	Miettinen	75	F					-	-	-	-		-													
10	2009	Miettinen	65	F					-	-	-	-		-													
11	2009	Miettinen	33	M					-	-	-	-		-													
12	2009	Miettinen	43	M					-	-	-	-		-													
13	2009	Miettinen	56	F					-	-	-	-		-													
14	2009	Miettinen	50	M					-	-	-	-		-													
15	2009	Miettinen	21	M					-	-	-	-		-													
16	2009	Miettinen	16	F					-	-	-	-		-													
17	2009	Miettinen	30	F					-	-	-	-		-													
18	2009	Miettinen	7	F					-	-	-	-		-													

19	2009	Pailoor	23	F		+	+			-	-	+/- (partial)		-													

20	2010	Sing	35	F	+		+	+		-	-	+	+	-	+				-	+	-	-	-	-			

21	2010	Takahashi	23	M		+		+		-	-			-		-	-	-					-			-	-

22	2010	Tan	34	M	<2%		+			-	-	+		-												-	-

23	2010	Wang	54	F	<1%	+	+			-	-			-													

24	2010	Cooper	64	M			+	+	-	-	-			-		-						-					

25	2011	Kim	52	M			+			-		+/- (partial)		-													

26	2012	Kang	47	M	6%		+			-	-	-		-		-	-										

27	2012	Kang	63	F			+			-	-	-		-		-										wt	wt

28	2012	Schulz	59	M	<2%		+			-	-	-		-		-	-						-	-			

29	2012	Cai	32	M			+	+		-	-			-													

30	2012	Cai	47	F			+	+		-	-			-													

31	2012	Wang	12	M			+	+	-	-	-			-													

32	2012	Miettinen	49	F		+	+			-											+ (variably)						

33	2012	Li	47	F	<1%		+			-	-		-	-								-	-				

34	2012	Bi	31	M			+	+								+											

35	2012	Bi	47	F			+	+																			

36	2012	Bi	42	F			+	+				+	+							+	+						

37	2014	Baek	38	F			+			-	-			-													

38	2014	Duckworth	16	F	1% (focally 8%)		+		-	-	-	-		-													

39	2014	Duckworth	11	F		+	+			-	-	+		-	+								-	-	-		

40	2014	Ikemura	27	F	Small Percentage		+			-	-	+/- (partial)							-	-	+						

41	2014	Lee	42	F			+	+	-	-	-	-		-								-					

42	2014	Li	32	M	<1%	+	+/- (partial)		-	-	-	+ (partial)	+ (partial)	-								-	-				

43	2014	Sakamoto	60	M			-		-	-	-			-							-						

44	2014	Li	73	F		+	+			-	-			-													

45	2014	Tian	64	M			+		-	-	-																

46	2015	Banerjee	19	F			-	-	-	-	-	-		-	-						-						

47	2015	Fassan	55	F	0%	+	+	+		-	+	-		-		-					-					-	-

48	2015	Lu	26	F		+	+		-	-	-																

49	2015	Ni	21	F	5%	+	+		-	-	-			-		-											

50	2015	Wei	50	F			+		-					-													

51	2015	Yue	34	M			+	+	-	-	-			-													

52	2015	Yue	50	F			+	+	-	-	-			-													

53	2015	Xu	50	F	<1%	+	+		-	-	-			-											-		

54	2016	Dixit	51	F	<1%		+			-	-		-	-								-	-				

55	2016	Inoue	36	F																							

56	2016	Jonaitis	28	F	40% (vascular endothelial Ki-67)		+				-	-		-													

57	2016	Kane	28	F			+		-	-	-			-							+/- (focal)	-		-			

58	2016	Morris	9	F			+		-	-	-	+/- (focal)		-	+			-			+/- (focal)		-				

59	2016	Nagahisa	39	M			+			-	-			-													

60	2016	Quero	47	M		+	+		-	-	-	+/- (partial)	+/- (partial)	-		-					+/- (partial)		-	+/- (focal)	-	wt	wt

61	2016	Spans	19	M			+			-	-	-		-									-				

62	2016	Spans	65	F			+			-	-	-		-		-							-				

63	2016	Spans	58	F			+			-	-	-		-		-							-				

64	2016	Spans	51	F			+					+/- (focal)		-													

65	2016	Spans	63	F			+					+/- (focal)		-													

66	2016	Spans	76	F			+/- (focal)					+															

67	2016	Spans	62	F								+/- (focal)		-													

68	2016	Spans	30	F			+					+															

69	2016	Spans	28	M			+				-	+/- (focal)		+									-				

70	2016	Spans	44	F							+/- (focal)	+		-													

71	2016	Spans	18	F			+/- (focal)					+/- (focal)															

72	2016	Spans	19	F			+			-	-	-		-		-											

73	2016	Spans	46	M			+				-	-		-													

74	2016	Spans	29	M			+			-	-	-		-									-				

75	2016	Spans	36	F			+			-	-	-		-													

76	2016	Spans	47	F			+			-	-	-		-													

77	2016	Li	11	M			+			-	-		+														

78	2016	Zhang	48	M		+	+		-	-	-			-													

79	2016	Li	44	F		+	-			-	+	-		-													

80	2017	Akai	55	M		+	-			-	+	-		-													

81	2017	Gonzalez-Cordero	37	M				+	-	-	-	+		-							+		-				

82	2017	Hu	26	M		+	+		-	-	+/- (negative/focal)	+/- (patchy, 5/10)	+/- (patchy, 6/10)	-					-	+ (patchy/ diffusely at nucleus, 6/10)	+ (patchy, 5/10)		-			wt (5/10)	wt (5/10)
83	2017	Hu	31	F		+	+		-	-				-					-				-				
84	2017	Hu	72	F		+	+		-	-				-					-				-				
85	2017	Hu	59	F		+	+		-	-				-					-				-				
86	2017	Hu	52	M		+	+		-	-				-					-				-				
87	2017	Hu	59	F		+	+		-	-				-					-				-				
88	2017	Hu	48	M		+	+		-	-				-					-				-				
89	2017	Hu	58	M		+	+		-	-				-					-				-				
90	2017	Hu	46	M		+	+		-	-				-					-				-				
91	2017	Hu	40	F		+	+		-	-				-					-				-				

92	2017	Kawara	66	M	2%		+ (focal)	+ (focal)	-	-	-	-	-	-	+ (focal)						-						

93	2017	Kim	51	M			+			-																	

94	2017	Liang	11	M			+ (focal)		-	-	-	-	+ (focal)	-	+ (focal)								-				

95	2017	Moris	63	M			+		-	-	-	-		-												wt	wt

96	2017	Qi	42	M	1%	+	+		-	-	-	+ (partial)	+ (partial)	-	+ (partial)	-					-			-			

97	2017	Qi	66	M	1%	+	+		-	-	-			-		-					-			-			

98	2017	Szurian	16	F			+		-	-	-	+ (focal)	+ (focal)	-							+/- (focal)	-	-			-	-

99	2017	Szurian	34	M			+		-	-	-	-	-	-							+/- (focal)	-	-				

100	2017	Wambura	41	F			+		-	-		-		-							-						

101	2017	Yang	50	F	3%	+	+		-	-	+	-		-		-		-			-	-	-				

102	2017	Zhou	51	M	1%		+		-	-	-	-		-										-		-	-

103	2018	Djurić	14	M			+		-	-	-	+ (focal)		-			-						-				

104	2018	Jang	47	F			+		-	-	-	+ (focal)		-		-					+ (focal)						

105	2018	Rohit	81	F			+ (patchy)		-	-	-	-		-										-			

106	2018	Wang	52	F	1%		+		-	-	-	-		+		-					+						

107	2018	Zhang	31	F	<5%		+		-	-	-	-		-		-		-			+ (partial)						

108	2019	Wu	59	F			+		-	-	-	+/- (focal)		-		-						-	-				

109	2019	Fukazawa	14	F	2%	+	+		-	-	-	-		-									-				

110	2019	Banerjee	65	M			+		-	-	-			-													

111	2019	Lai	21	F			+			-	-	-		-													

112	2019	Lai	42	F					-	-	-	-		-													

113	2019	Lai	79	F	30%		+		-	-	-	+/- (focal)		-													

114	2019	Lai	33	M			+			-	-	+/- (focal)		-													

115	2019	Lai	60	M			+		-	-	-	+ (focal)		-													

116	2019	Lai	77	M			+		-	-	-																

117	2019	Lai	45	M			+		-	-	-																

118	2019	Li	5	M		+	+		-	-	-	-		-								-					

^†^Abbreviations: Dx, diagnosis; Vim, vimentin; Cald, caldesmon; Calp, calponin; NF, neurofilament; CK, cytokeratin; *β*-C, *β*-catenin; SNP, synaptophysin; wt, wild type.
